# Age and Age-related Network Diversity as Predictors of Stress Reduction Among Cancer Survivors and Care Partners

**DOI:** 10.1093/geroni/igaf122.567

**Published:** 2025-12-31

**Authors:** Kristin Cloyes, Jia-Wen Guo, Kelly Mansfield, Sarah Wawrzynski, Maija Reblin

**Affiliations:** Oregon Health & Science University, Portland, Oregon, United States; University of Utah College of Nursing, Salt Lake City, Utah, United States; University of Utah College of Nursing, Salt Lake City, Utah, United States; Nemours Children’s Health, Wilmington, Delaware, United States; University of Vermont, Burlington, Vermont, United States

## Abstract

Social support, an established driver of health outcomes, reflects resources conferred by social relationships that shift across life course, circumstances, and events. For example, structural social network characteristics shape support resource access and quality. Older adults may consolidate networks, prioritizing strong emotional ties, while people with chronic illness and caregivers experience shrinking networks. Broader social context like the pandemic can intensify these effects. We performed secondary analysis of cancer survivor and care partner social network (baseline) and perceived stress data (baseline and 3 months) collected October 2020 through April 2021 (N = 64, 32 survivor/partner dyads, Mean age = 43.3, SD = 17.18). Binary logistic regression examined whether network structure (density, modularity, age diversity) predicted positive or negative changes in stress, and if this differed by age. Density and modularity were non-significant and removed to improve model stability. The simplified model, retaining age and age-related diversity, showed that participants’ age was significantly associated with stress reduction (B = -0.046, p = .016; Exp(B) = 0.955); for each additional year of age, the odds of decreased stress rose by 5.1%. Age-related network diversity demonstrated a trend toward reduced stress reduction (B = -0.092, p = .132; Exp(B) = 0.912). Our prior work found that age diversity positively predicted support, yet older participants had less age-diverse networks. Current findings suggest age-diverse networks may moderate stress during stressful life events, possibly due to differences in network function like availability of resources. Future research focusing on support availability within networks over time could address this gap.

